# Supplementary material for: Altitudinal gradients in Magellanic sub-Antarctic lagoons: the effect of elevation on freshwater macroinvertebrate diversity and distribution
Source: PeerJ. 2019 Jul 29;7:e7128. doi: 10.7717/peerj.7128 (PMC6673463; doi:10.7717/peerj.7128)
Supplement: Supplemental Information 1 — Supplementary table 1. Abiotic parameters. Supplementary table 2. Taxonomic macroinvertebrate list.Supplementary table 3. Analyses of diversity metrics. Supplementary table 4. Post-hoc comparisons of diversity metrics. Supplementary table 5. Analyses of macroinvertebrate composition. Supplementary table 6. Post-hoc comparisons of macroinvertebrate composition.Supplementary table 7. Analyses of macroinvertebrate functional feeding groups. Supplementary table 8. Post-hoc comparisons of macroinvertebrate functional feeding groups. [file peerj-07-7128-s001.docx]

**SUPPLEMENTARY TABLES**

Supplementary table 1. Abiotic parameters. Wilcoxon post hoc pairwise comparisons for abiotic parameters measured at sampling sites in the Róbalo river watershed, Navarino Island, Chile. Values with asterisks represent significant differences (p < 0.05).

| **Groups** | **Conductivity (μS/cm)** | **pH** | **Temperature (°C)** |
| --- | --- | --- | --- |
| 20 - 250 | <0.0001* | <0.0001* | 0.0205* |
| 20 - 480 | <0.0001* | <0.0001* | <0.0001* |
| 20 - 700 | <0.0001* | <0.0001* | <0.0001* |
| 250 - 480 | <0.0001* | 0.0374* | <0.0001* |
| 250 - 700 | <0.0001* | 0.1569 | <0.0001* |
| 480 - 700 | <0.0001* | 0.0208* | <0.0001* |

Supplementary table 2. Taxonomic macroinvertebrate list. List of macroinvertebrates associated to lagoons in the present study. Each elevation level corresponds to a lagoon located in the Róbalo river watershed, Navarino Island, Chile. Abbreviations, FFG = Functional feeding groups, C-G = collector gatherers, C-fil = collector filterers, C-pred = collector predators, Herb = herbivores, Pred = predators, Sh = shredders. The “x” stands for presence at a given elevation.

|  |  |  |  |  | **Elevation (m.a.s.l.)** | | | |
| --- | --- | --- | --- | --- | --- | --- | --- | --- |
| **Class** | **Order** | **Family** | **Genus/ species/ morpho-species** | **FFG** | **20** | **250** | **480** | **700** |
| CLITELLATA | HIRUDINAE | Glossiphoniidae | Glos-morph1 | Pred | x | x | x | x |
|  |  |  | Glos-morph2 | Pred |  | x |  |  |
|  | OLIGOCHAETA |  | Oligo-morph1 | C-G | x | x | x | x |
| MALACOSTRACA | AMPHIPODA | Hyalellidae | *Hyalella sp.* | C-G | x | x | x | x |
| BRANCHIOPODA | CLADOCERA | Daphniidae | Daph-morph1 | C-fil | x |  |  |  |
| INSECTA | COLEOPTERA | Dytiscidae | *Lancetes angusticollis* | Pred | x | x | x |  |
|  |  |  | *Liodessus* | Pred | x |  |  |  |
|  | DIPTERA | Ceratopogonidae | Cerato-morf1 | C-pred | x | x |  |  |
|  |  | Chironomidae | Chiro-morph1 | C-G | x | x | x | x |
|  |  |  | Chiro-morph2 | C-G | x | x |  | x |
|  |  |  | Chiro-morph3 | C-G | x | x | x |  |
|  |  |  | Chiro-morph4 | C-G | x |  |  |  |
|  |  |  | Chironom-morph1 | C-G | x |  |  |  |
|  |  |  | Orth-morph1 | C-G | x |  |  |  |
|  |  |  | Tany-morph1 | Pred | x |  |  |  |
|  |  | Empididae | *Hemerodromia* | Pred | x | x | x |  |
|  |  | Tipulidae | *Hexatoma* | Pred |  | x |  |  |
|  | EPHEMEROPTERA | Leptophlebiidae | *Meridialaris chiloeensis* | Sh |  | x |  |  |
|  |  | Nessameletidae | *Metamonius anceps* | Sh |  |  | x |  |
|  | HEMIPTERA | Corixidae | *Sigara* sp. | Herb | x |  |  |  |
|  | ODONATA | Aeshnidae | *Rhionaeschna variegata* | Pred | x |  |  |  |
|  | PLECOPTERA | Gripopterygidae | *Aubertoperla kuscheli* | Sh |  |  |  | x |
|  | TRICHOPTERA | Limnephilidae | *Austrocosmoecus sp.* | Herb |  | x | x |  |
|  |  |  | *Monocosmoecus hyadesi* | Herb |  | x |  |  |
|  |  |  | *Verger*-morph1 | Herb |  | x | x |  |
|  |  |  | *Verger*-morph2 | Herb |  | x |  |  |
| BIVALVIA | VENEROIDA | Sphaeriidae | *Pisidium magellanicum* | C-fil | x |  | x |  |
| GASTROPODA | HYGROPHILA | Lymnaeidae | *Pectinidens diaphanum* | Herb |  | x |  |  |

Supplementary table 3. Analyses of diversity metrics. Univariate Permutation analysis (PERMANOVA) of diversity metrics from sampled lagoons of the Róbalo river watershed, Navarino Island, Chile. The design was factorial type, considering Elevation and Habitat. Data was based on Euclidean distances and 4999 permutations were performed. Values with asterisks indicate significant differences (p < 0.05). Abbreviations, Elev = Elevation, Hab = Habitat type.

| **Metric** | **Source** | **df** | **SS** | **MS** | **F** | ***p*** |
| --- | --- | --- | --- | --- | --- | --- |
| Richness | Elev | 3 | 295.23 | 98.41 | 39.695 | 0.0002* |
|  | Hab | 3 | 38.729 | 12.91 | 5.2073 | 0.0056* |
|  | Elev x Hab | 9 | 33.188 | 3.6875 | 1.4874 | 0.1958 |
|  | Res | 32 | 79.333 | 2.4792 |  |  |
|  |  |  |  |  |  |  |
| Abundance | Elev | 3 | 1250200 | 416730 | 56.544 | 0.0002* |
|  | Hab | 3 | 277280 | 92422 | 12.54 | 0.0002* |
|  | Elev x Hab | 9 | 697450 | 77495 | 10.515 | 0.0002* |
|  | Res | 32 | 2460800 | 7370 |  |  |
|  |  |  |  |  |  |  |
| Shannon | Elev | 3 | 8.0297 | 2.6766 | 31.566 | 0.0002* |
|  | Hab | 3 | 0.86498 | 0.28833 | 3.4004 | 0.0294* |
|  | Elev x Hab | 9 | 1.5561 | 0.1729 | 2.0391 | 0.0628 |
|  | Res | 32 | 2.7134 | 0.084793 |  |  |
|  |  |  |  |  |  |  |
| Evenness | Elev | 3 | 1.3023 | 0.4341 | 25.478 | 0.0002* |
|  | Hab | 3 | 0.30455 | 0.10152 | 5.9582 | 0.0026* |
|  | Elev x Hab | 9 | 0.6452 | 0.071689 | 4.2076 | 0.0018* |
|  | Res | 32 | 0.54522 | 0.017038 |  |  |
|  |  |  |  |  |  |  |
| Hill´s N1 | Elev | 3 | 54.426 | 18.142 | 18.156 | 0.0002* |
|  | Hab | 3 | 5.1652 | 1.7217 | 1.7231 | 0.1784 |
|  | Elev x Hab | 9 | 10.757 | 1.1953 | 1.1962 | 0.3234 |
|  | Res | 32 | 31.974 | 0.9992 |  |  |

Supplementary table 4. Post-hoc comparisons of diversity metrics. P-values for post-hoc comparisons conducted after PERMANOVA on diversity metrics. Values with asterisks indicate significant differences (p < 0.05). Abreviations: Elev = Elevation, Hab = Habitat, rock = rock bottoms, sub veg = submerged vegetation, aq moss = aquatic mosses.

| **Richness** |  |  |  |  |
| --- | --- | --- | --- | --- |
| **Groups** | ***p*** |  | **Groups** | ***p*** |
| 20 - 250 | 0.0062* |  | rock – sub veg | 0.0786 |
| 20 - 480 | 0.0002* |  | rock – debris | 0.5836 |
| 20 - 700 | 0.0002* |  | rock - aq moss | 0.0088* |
| 250 - 480 | 0.0566 |  | sub veg - debris | 0.0776 |
| 250 - 700 | 0.0002* |  | sub veg - aq moss | 0.0784 |
| 480 - 700 | 0.0004* |  | debris - aq moss | 0.0052* |

| **Abundance** |  |  |  |  |
| --- | --- | --- | --- | --- |
| **Groups** | ***p*** |  | **Groups** | ***p*** |
| 20 - 250 | 0.0002* |  | rock - sub veg | 0.0014* |
| 20 - 480 | 0.0002* |  | rock – debris | 0.0002* |
| 20 - 700 | 0.0002* |  | rock - aq moss | 0.0002* |
| 250 - 480 | 0.0002* |  | sub veg - debris | 0.7976 |
| 250 - 700 | 0.0004* |  | sub veg - aq moss | 0.2974 |
| 480 - 700 | 0.0006* |  | debris - aq moss | 0.2098 |

| **Groups** | **rock** | **sub veg** | **debris** | **aq moss** |  | **Groups** | **20** | **250** | **480** | **700** |
| --- | --- | --- | --- | --- | --- | --- | --- | --- | --- | --- |
| 20 - 250 | 0.1662 | 0.004* | 0.0002* | 0.037* |  | rock - sub veg | 0.0072* | 0.72 | 0.7952 | 0.156 |
| 20 - 480 | 0.1662 | 0.0046* | 0.0038* | 0.2456 |  | rock - debris | 0.0018* | 0.3274 | 0.0002* | 0.0554 |
| 20 - 700 | 0.2552 | 0.007* | 0.0006* | 0.0752 |  | rock - aq moss | 0.0892 | 0.8914 | 0.0024* | 0.0928 |
| 250 - 480 | 0.5606 | 0.9032 | 0.0002* | 0.0016* |  | sub veg - debris | 0.0708 | 0.1966 | 0.0006* | 0.5178 |
| 250 - 700 | 0.3908 | 0.0766 | 0.0056* | 0.0274* |  | sub veg - aq moss | 0.0902 | 0.5882 | 0.0036* | 0.7564 |
| 480 - 700 | 0.6064 | 0.0972 | 0.001* | 0.024* |  | debris - aq moss | 0.601 | 0.2252 | 0.0192* | 0.7448 |

| **Shannon** |  |  |  |  |
| --- | --- | --- | --- | --- |
| **Groups** | ***p*** |  | **Groups** | ***p*** |
| 20 - 250 | 0.1856 |  | rock - sub veg | 0.7846 |
| 20 - 480 | 0.0006* |  | rock - debris | 0.09 |
| 20 - 700 | 0.0002* |  | rock - aq moss | 0.2542 |
| 250 - 480 | 0.0032* |  | sub veg - debris | 0.068 |
| 250 - 700 | 0.0002* |  | sub veg - aq moss | 0.1158 |
| 480 - 700 | 0.0002* |  | debris - aq moss | 0.0036* |

| **Pielou** |  |  |  |  |
| --- | --- | --- | --- | --- |
| **Groups** | ***p*** |  | **Groups** | ***p*** |
| 20 - 250 | 0.0016* |  | rock - sub veg | 0.0702 |
| 20 - 480 | 0.7972 |  | rock - debris | 0.0004* |
| 20 - 700 | 0.0002* |  | rock - aq moss | 0.5876 |
| 250 - 480 | 0.0016* |  | sub veg - debris | 0.0224* |
| 250 - 700 | 0.0002* |  | sub veg - aq moss | 0.2664 |
| 480 - 700 | 0.001* |  | debris - aq moss | 0.006* |

| **Groups** | **rock** | **sub veg** | **debris** | **aq moss** |  | **Groups** | **20** | **250** | **480** | **700** |
| --- | --- | --- | --- | --- | --- | --- | --- | --- | --- | --- |
| 20 - 250 | 0.5282 | 0.0082* | 0.0162* | 0.4094 |  | rock - sub veg | 0.0328* | 0.961 | 0.5176 | 0.2952 |
| 20 - 480 | 0.2128 | 0.0498* | 0.0244* | 0.0246* |  | rock - debris | 0.3352 | 0.3516 | 0.0092* | 0.0002* |
| 20 - 700 | 0.0066* | 0.202 | 0.0016* | 0.2954 |  | rock - aq moss | 0.6312 | 0.7718 | 0.046* | 0.6828 |
| 250 - 480 | 0.6566 | 0.8744 | 0.002* | 0.0532 |  | sub veg - debris | 0.158 | 0.1042 | 0.013* | 0.0606 |
| 250 - 700 | 0.037* | 0.0116* | 0.0004* | 0.188 |  | sub veg - aq moss | 0.0134* | 0.6624 | 0.0978 | 0.3892 |
| 480 - 700 | 0.016* | 0.0264* | 0.0348* | 0.829 |  | debris - aq moss | 0.1714 | 0.4648 | 0.0126* | 0.0698 |

| **N1** |  |
| --- | --- |
| **Groups** | ***p*** |
| 20 - 250 | 0.1436 |
| 20 - 480 | 0.0004* |
| 20 - 700 | 0.0002* |
| 250 - 480 | 0.0028* |
| 250 - 700 | 0.0006* |
| 480 - 700 | 0.0004* |

Supplementary table 5. Analyses of macroinvertebrate composition. Multivariate Permutation analysis (PERMANOVA) of freshwater macroinvertebrate composition from sampled lagoons of the Róbalo river watershed, Navarino Island, Chile. The design was factorial type, considering Elevation and Habitat type. Data was based on Bray-Curtis similarity and 4999 permutations were performed. Values with asterisks indicate significant differences (p < 0.05). Abbreviations, Elev = Elevation, Hab = Habitat type.

| **Source** | **Df** | **SS** | **MS** | **F** | ***p*** |
| --- | --- | --- | --- | --- | --- |
| Elev | 3 | 36341 | 12114 | 20.149 | 0.0002* |
| Hab | 3 | 5907.7 | 1969.2 | 3.2755 | 0.0002* |
| Elev x Hab | 9 | 14732 | 1636.9 | 2.7226 | 0.0002* |
| Res | 32 | 19239 | 601.21 |  |  |

Supplementary table 6. Post-hoc comparisons of macroinvertebrate composition**.** P-values for post-hoc comparisons conducted after PERMANOVA on macroinvertebrate composition. Values with asterisks indicate significant differences (p < 0.05). Abbreviations, Elev = Elevation, Hab = Habitat, rock = rock bottoms, sub veg = submerged vegetation, aq moss = aquatic mosses.

| **Groups** | ***p*** |  | **Groups** | ***p*** |
| --- | --- | --- | --- | --- |
| 20 - 250 | 0.0002* |  | rock – sub veg | 0.1376 |
| 20 - 480 | 0.0002* |  | rock - debris | 0.0018* |
| 20 - 700 | 0.0002* |  | rock - aq moss | 0.0014* |
| 250 - 480 | 0.0002* |  | sub veg - debris | 0.0852 |
| 250 - 700 | 0.0002* |  | sub veg - aq moss | 0.0102* |
| 480 - 700 | 0.0002* |  | debris - aq moss | 0.0006* |

| **Groups** | **rock** | **sub veg** | **debris** | **aq moss** |  | **Groups** | **20** | **250** | **480** | **700** |
| --- | --- | --- | --- | --- | --- | --- | --- | --- | --- | --- |
| 20 - 250 | 0.0494* | 0.0079* | 0.0172* | 0.0019* |  | rock - veg sum | 0.0147* | 0.1934 | 0.3784 | 0.7402 |
| 20 - 480 | 0.009* | 0.0317* | 0.0032* | 0.0017* |  | rock - debris | 0.0425* | 0.2052 | 0.0524 | 0.0474* |
| 20 - 700 | 0.0091* | 0.0021* | 0.0016* | 0.0014* |  | rock - aq moss | 0.0146* | 0.0828 | 0.0549 | 0.0779 |
| 250 - 480 | 0.3329 | 0.1347 | 0.0173* | 0.005* |  | veg sum - debris | 0.2079 | 0.3241 | 0.0968 | 0.0518 |
| 250 - 700 | 0.1254 | 0.0223* | 0.0162* | 0.0145* |  | veg sum - aq moss | 0.0512* | 0.1393 | 0.091 | 0.0347* |
| 480 - 700 | 0.0879 | 0.2765 | 0.0015 | 0.0067* |  | debris - aq moss | 0.3017 | 0.062 | 0.0344* | 0.0155* |

Supplementary table 7. Analyses of macroinvertebrate functional feeding groups. Multivariate Permutation analysis (PERMANOVA) of functional feeding groups (FFG) composition from sampled lagoons of the Róbalo river watershed, Navarino Island, Chile. The design was factorial type, considering Elevation and Habitat. Data was based on Bray-Curtis similarity and 4999 permutations were performed. Values with asterisks indicate significant differences (p < 0.05). Abbreviations, Elev = Elevation, Hab = Habitat type.

| **Source** | **df** | **SS** | **MS** | **F** | ***p*** |
| --- | --- | --- | --- | --- | --- |
| Elev | 3 | 20390 | 6796.6 | 25.168 | 0.0002* |
| Hab | 3 | 3752.6 | 1250.9 | 4.6319 | 0.0002* |
| Elev x Hab | 9 | 5176.5 | 575.16 | 2.1298 | 0.0032* |
| Res | 32 | 8641.7 | 270.05 |  |  |

Supplementary table 8. Post-hoc comparisons of macroinvertebrate functional feeding groups. P-values for post-hoc comparisons conducted after PERMANOVA on freshwater macroinvertebrate functional feeding groups (FFG). Values with asterisks indicate significant differences (p < 0.05). Abbreviations, Elev = Elevation, Hab = Habitat, rock = rock bottoms, sub veg = submerged vegetation, aq moss = aquatic mosses.

| **Groups** | ***p*** |  | **Groups** | ***p*** |
| --- | --- | --- | --- | --- |
| 20 - 250 | 0.0002* |  | rock – sub veg | 0.0544 |
| 20 - 480 | 0.0002* |  | rock – debris | 0.0002* |
| 20 - 700 | 0.0002* |  | rock - aq moss | 0.0002* |
| 250 - 480 | 0.0002* |  | sub veg - debris | 0.3382 |
| 250 - 700 | 0.0002* |  | sub veg - aq moss | 0.0182* |
| 480 - 700 | 0.0002* |  | debris - aq moss | 0.045* |

| **Groups** | **rock** | **veg sum** | **debris** | **aq moss** |  | **Groups** | **20** | **250** | **480** | **700** |
| --- | --- | --- | --- | --- | --- | --- | --- | --- | --- | --- |
| 20 - 250 | 0.0654 | 0.0296* | 0.0152* | 0.005* |  | rock - sub veg | 0.1234 | 0.2414 | 0.5334 | 0.5324 |
| 20 - 480 | 0.1858 | 0.0308* | 0.0092* | 0.0008* |  | rock - debris | 0.057 | 0.073 | 0.0462* | 0.1754 |
| 20 - 700 | 0.0186* | 0.0076* | 0.0016* | 0.0002* |  | rock - aq moss | 0.0148* | 0.0266* | 0.0416* | 0.2086 |
| 250 - 480 | 0.6043 | 0.1616 | 0.029* | 0.0062* |  | sub veg - debris | 0.5551 | 0.3064 | 0.1054 | 0.3936 |
| 250 - 700 | 0.0376* | 0.0254* | 0.0048* | 0.0026* |  | sub veg - aq moss | 0.1834 | 0.1342 | 0.0924 | 0.4174 |
| 480 - 700 | 0.6124 | 0.1204 | 0.001* | 0.0016* |  | debris - aq moss | 0.4672 | 0.0894 | 0.0192* | 0.7218 |
